# Supplementary material for: Activity of Antioxidant Enzymes and Their Association with Lipid Profile in Mexican People without Cardiovascular Disease: An Analysis of Interactions
Source: Int J Environ Res Public Health. 2018 Nov 28;15(12):2687. doi: 10.3390/ijerph15122687 (PMC6313725; doi:10.3390/ijerph15122687)
Supplement: Supplementary file 1 [file ijerph-15-02687-s001.zip › ijerph-384772-supplementary-edited.pdf]

Table S1. Analysis of the activity of antioxidant enzymes and FRAP according to factors associated with dyslipidemia or antioxidant enzyme activity.

Factor		PON1 Activity (U/ml)	SOD1 Activity (U/mg)	Catalase Activity (k/mg)	Milliequivalents of Trolox (μM)	Ceruloplasmin activity (U/ml)
Physical activity	Low	273.13 ± 114.89	10.71 ± 2.87	0.38 ± 0.20	960.36 ± 356.28	2.26 ± 0.36
	Moderate	284.08 ±144.66	10.87 ± 2.63	0.32 ±0.17	992.15 ± 361.08	2.29 ± 0.31
	High	264.90 ± 130.43	11.26 ± 2.99	0.33 ± 0.18	1054.39 ± 405.82	2.29 ± 0.32
	p-value	0.245	0.156	0.064	0.070	0.774
Smoking habit	Nonsmoker	274.84 ± 135.41	11.10 ± 2.89	0.33 ± 0.18	980.96 ± 363.89	2.28 ± 0.32
	Current smoker	281.32 ± 134.29	11.13 ± 2.84	0.32 ± 0.17	1073.15 ± 446.22	2.31 ± 0.33
	Ex-smoker	260.87 ±136.27	10.69 ± 2.63	0.32 ± 0.17	1094.86 ± 359.11	2.29 ± 0.33
	p-value	0.504	0.385	0.625	0.005	0.601
Gender	Women	268.98 ± 133.42	11.34 ± 3.03	0.32 ± 0.18	921.37 ± 326.65	2.28 ± 0.30
	Men	282.05 ± 138.26	10.53 ± 2.39	0.34 ± 0.17	1184.91 ± 416.27	2.30 ± 0.36
	p-value	0.244	0.001	0.028	<0.001	0.354
Alcohol consumer	Yes	271.05 ± 133.26	10.96 ± 2.91	0.32 ± 0.17	1030.68 ± 393.38	2.27 ± 0.30
	No	280.82 ± 140.39	11.25 ± 2.63	0.35 ± 0.18	987.79 ± 356.98	2.30 ± 0.33
	p-value	0.417	0.255	0.145	0.209	0.341
Age	Correlation	-0.32	0.040	0.034	-0.089	0.001
	p-value	0.430	0.315	0.393	0.025	0.970
Caloric intake	Correlation	0.081	-0.091	0.059	0.131	0.021
	p-value	0.042	0.022	0.138	0.001	0.597

Categorical variables with more than two categories were compared using one-way ANOVA or the Kruskal–Wallis test (according to the fulfillment of the correspondent assumptions), whereas those with two categories were compared by Student's *t*-test or the Mann–Whitney U-test (according to the fulfillment of the correspondent assumptions). For continuous variables a Pearson correlation test was used without adjustment for other covariates.

Table S2. Association between antioxidant enzyme activities and FRAP.

Parameter		PON1 Activity (U/ml)	SOD1 Activity (U/mg)	Catalase Activity (k/mg)	Milliequivalents of Trolox (μM)	Ceruloplasmin activity (U/ml)
PON1 activity	Correlation	-	-.095	.058	-.192	-.080
(U/ml)	*p*-value	-	.018	.151	.000	.046
SOD1 activity	Correlation	-.095	-	.267	.105	.114
(U/mg)	*p*-value	.018	-	.000	.009	.004
Catalase activity	Correlation	.058	.267	-	-.452	-.028
(k/mg)	*p*-value	.151	.000	-	.000	.485
Milliequivalents	Correlation	-.192	.105	-.452	-	.022
of Trolox (μM)	*p*-value	.000	.009	.000	-	.577
Ceruloplasmin	Correlation	-.080	.114	-.028	.022	-
activity (U/ml)	*p*-value	.046	.004	.485	.577	-

The correlations are the results from a partial correlation analysis adjusted by age, gender, tobacco smoking, alcohol use, physical activity, and caloric intake.

Table S3. Results from the linear regression models considering the main effects of the components of lipid profiles and their association with the activity of superoxide dismutase-1, catalase, and FRAP.

Parameter	Term in the Equation	Unadjusted β Coefficient (95%CI)	Unadjusted Standardized β Coefficient	*p*-Value	Adjusted β Coefficient (95%CI)	Adjusted Standardized β Coefficient	*p*-Value
Superoxide dismutase-1	↑TG	0.12 (-1.09, 1.33)	0.020	0.845	0.56 (-0.66, 1.79)	0.095	0.369
	↑LDLC	0.20 (-0.55, 0.96)	0.034	0.599	0.14 (-0.63, 0.91)	0.024	0.718
	↓HDLC	-0.08 (-0.76, 0.61)	-0.013	0.826	-0.17 (-0.85, 0.51)	-0.029	0.629
	↑TGx↑LDLC	-0.87 (-2.42, 0.68)	-0.120	0.272	-0.95 (-2.49, 0.59)	-0.132	0.227
	↑TGx↓HDLC	-0.101 (-1.56, 1.36)	-0.015	0.893	-0.35 (-1.81, 1.11)	-0.047	0.640
	↑LDLCx↓HDLC	0.72 (-0.69, 2.13)	0.088	0.319	0.73 (-0.67, 2.13)	0.090	0.306
	↑TGx↑LDLCx↓HDLC	-0.39 (-2.56, 1.78)	-0.041	0.726	-0.32 (-2.45, 1.84)	-0.033	0.771
Log catalase activity	↑TG	0.03 (-0.08, 0.12)	0.051	0.627	-0.01(-0.11, 0.09)	-0.013	0.809
	↑LDLC	-0.01 (-0.07,0.05)	-0.018	0.778	-0.02 (-0.08, 0.05)	-0.030	0.651
	↓HDLC	-0.04 (-0.09, 0.02)	-0.077	0.207	-0.04 (-0.09, 0.02)	-0.077	0.209
	↑TGx↑LDLC	0.01 (-0.12, 0.14)	0.016	0.883	0.03 (-0.10, 0.16)	0.049	0.656
	↑TGx↓HDLC	-0.00 (-0.13, 0.12)	-0.008	0.944	0.01 (-0.11, 0.13)	0.023	0.837
	↑LDLCx↓HDLC	0.05 (-0.07, 0.16)	0.069	0.437	0.05 (-0.07, 0.17)	0.076	0.389
	↑TGx↑LDLCx↓HDLC	-0.06 (-0.24, 0.12)	-0.073	0.531	-0.07 (-0.25, 0.11)	-0.092	0.428
Log ferric-reducing ability	↑TG	0.04 (-0.02, 0.11)	0.136	0.183	0.02 (-0.05, 0.08)	0.048	0.624
	↑LDLC	0.01 (-0.03, 0.05)	0.028	0.665	0.03 (-0.01, 0.07)	0.093	0.132
	↓HDLC	0.01 (-0.03, 0.05)	0.030	0.615	0.03 (-0.10, 0.06)	0.081	0.153
	↑TGx↑LDLC	0.03 (-0.06,0.11)	0.065	0.547	0.01 (-0.07, 0.09)	0.022	0.831
	↑TGx↓HDLC	0.02 (-0.06,0.10)	0.066	0.543	0.04 (-0.04, 0.11)	0.103	0.315
	↑LDLCx↓HDLC	0.02 (-0.06, 0.10)	0.044	0.612	0.01 (-0.06, 0.09)	0.031	0.702
	↑TGx↑LDLCx↓HDLC	-0.08 (-0.20, 0.04)	-0.149	0.191	-0.07 (-0.18, 0.04)	-0.139	0.198

The adjusted β coefficient resulted from the linear models adjusted for all the covariates (age, gender, tobacco smoking, alcohol use, physical activity, and caloric intake).
